# Large volume scanning laser induced fluorescence measurement of a bluff-body stabilised flame in an annular combustor

**DOI:** 10.1007/s00348-022-03406-9

**Published:** 2022-03-23

**Authors:** Dirren Govender, Samuel Wiseman, James R. Dawson, Nicholas A. Worth

**Affiliations:** Department of Energy and Process Engineering, Norwegian University of Science and Technology, Kolbjørn Hejes vei 2, Trondheim, 7491 NO Norway

**Keywords:** Flame surface density, Planar laser-induced fluorescence, Scanning, Flame structure

## Abstract

**Abstract:**

This study outlines a variant of three-dimensional OH planar laser-induced fluorescence and its application in characterising a single bluff body stabilised flame inside a 12 burner annular combustor. In this variant of the method a relatively large volume was scanned slowly in order to calculate the full three-dimensional Flame Surface Density (FSD) distribution. The method used a combination of two scanning directions to overcome bias errors associated with laser sheet positions close to the flame edges. The source of this bias error was confirmed numerically through a complimentary synthetic PLIF study, which was also used to refine the experimental setup. The bias error resulted in a reduction of FSD magnitude, although the method was still capable of capturing the flame structure. This was demonstrated by comparing the reconstructions from the two independent scan directions. Combining the data from both directions overcame the bias, and allowed flame asymmetry due to the confinement to be assessed. The FSD was used to determine the heat release rate of the flame with varying local azimuthal angle for different downstream regions. This highlighted the highly asymmetric structure, produced by the asymmetric confinement.

**Graphical abstract:**

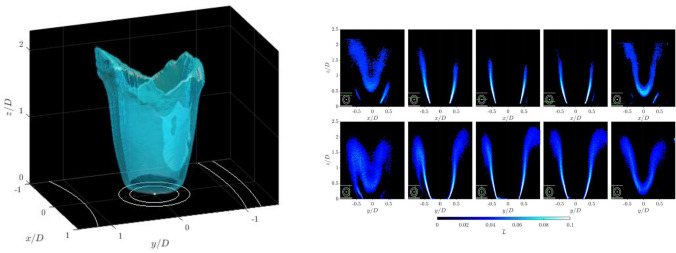

## Introduction

The move to efficient low emission combustion systems requires a better understanding of the physics of reacting flows. In modern gas turbine engines, flames are often confined within annular combustion chambers. However, a common simplification is to investigate the behaviour of single isolated flames in axisymmetric geometry. The characteristics of single flames have been extensively studied through the heat release rate distribution, which is often captured using two-dimensional (2D) planar or integrated line of sight techniques, such as for example OH planar laser-induced fluorescence (PLIF)  (Hanson et al. 1990; Pun et al. 2003; Cho et al. 2013; Mulla et al. 2016), or OH chemiluminescence (OH$^*$)  (He et al. 2019; Sardeshmukh et al. 2017; Doan and Swaminathan 2019) imaging, respectively. Under an assumption of axisymmetry such methods are capable of describing the full three-dimensional (3D) distribution of the heat release rate, which is only strictly true in turbulent flames when describing the time-average or some phase-average distribution. However, such assumptions are no longer applicable even in a time average sense when the local flow symmetry of the flame is broken by the confinement; such as that which occurs in annular geometry (Fanaca et al. 2010; Worth and Dawson 2013b). Therefore, the current investigation focuses on a fully three-dimensional measurement technique, capable of characterising the asymmetric heat release rate structure of a flame confined in an annular combustion chamber.

The most widely used method of resolving the full 3D heat release rate field is Computed Tomography of Chemiluminescence (CTC) (Worth and Dawson 2013a; Cai et al. 2013; Dreizler et al. 2018; Ishino and Ohiwa 2005; Floyd and Kempf 2011). CTC uses the principles of tomography to reconstruct the 3D distribution of chemiluminescence based on multiple integrated line of sight measurements taken by multiple cameras at different viewing angles (Floyd and Kempf 2011). In these studies chemiluminescence from a limited spectral band is used as a proxy for the heat release rate. The approach requires optical access across a large range of viewing angles, and the reconstruction accuracy can be highly dependent on the number of views and angles used. Therefore, in order to obtain a sufficient number of views either a large number of cameras can be employed (Unterberger et al. 2018; Meyer et al. 2016; Gilabert et al. 2007; Ishino et al. 2008), views can be gathered by optic fibres and collected on a single camera (Liu et al. 2019; Yu et al. 2020; Moinul Hossain et al. 2012; Mohamad et al. 2006), or a single camera can be re-positioned to capture multiple views in order to capture the time average or phase average response (Floyd and Kempf 2011; Worth and Dawson 2012; Wiseman et al. 2017).

It is common to use a thin laser light sheet to perform PLIF measurements in order to capture the distribution of fluorescence intensity in a plane of interest (Wabel et al. 2018). However, a recent extension of this standard approach has been used to capture full 3D volumes, by using a thick laser light sheet to fluoresce a volume of interest in a method known as Volumetric LIF (VLIF) (Xu et al. 2017; Meyer et al. 2016; Jiang et al. 2016; Wu et al. 2015; Ma et al. 2017; Halls et al. 2016). In this method, fluorescence emissions are captured by multiple cameras with a range of viewing angles, and these views are used to tomographically reconstruct a three-dimensional distribution of the targeted species. A comprehensive study by Xu et al. (2017) outlines the fundamental capabilities and limitations of the VLIF technique, where the authors used a numerical study to show that the reconstruction of symmetric flames is possible with high accuracy. However the technique is constrained by laser power, resulting in relatively thin volumes of interest. Therefore, the technique is often used to highlight only small regions of interest. While such an approach would in principle be able to effectively highlight a single flame in an annular rig, capturing the entire three-dimensional structure of a flame may still be difficult since very high powered lasers and multiple cameras would be required.

An alternative method of capturing three-dimensional structures using LIF is realised by combining a PLIF setup with scanning optics (Kychakoff et al. 1987; Cho et al. 2014; Miller et al. 2014; Wellander et al. 2014; Yip et al. 1986, 1994; Li et al. 2017; Weinkauff et al. 2015). In Scanning-PLIF a three-dimensional flow can be resolved by rapidly scanning a laser sheet through a volume of interest, and reconstructing the three-dimensional distribution by combining data from planar slices. In this approach the technique is constrained by the scanning speed, which is required to be much faster than the flow speed, in order to effectively *freeze* the flow (Weinkauff et al. 2015). The moderate to high velocities present in turbulent reacting flows require very high-speed scanning optics, which operate at frequencies of order  0.1 to 3 MHz (Cho et al. 2014). Such scanning speeds are not possible with current scanning devices and a trade-off between scan depth and speed is often a limitation of such techniques  (Römer and Bechtold 2014). Therefore, in comparison with mechanical scanning devices typically used in Scanning-PIV experiments for low speed flows (Lawson and Dawson 2014; Hori and Sakakibara 2004; Casey et al. 2013), high speed devices such as acousto-optic deflectors that can operate at a scanning rates of order 100 kHz have been used to scan reacting flows such as a lifted turbulent jet flame (Li et al. 2017), however the final scanning rate may also be limited by the laser or imaging systems. The scanning speed limitation again typically restricts such studies to reasonable small number of slices, and therefore to volumes of interest which are relatively thin.

In the current paper a new variant of the Scanning-PLIF approach is introduced, which aims to reconstruct the heat release rate (HRR) over a much larger volume of interest than previous approaches. This is achieved by focusing on characterising the time average structure of a reacting flow, meaning the restrictions on scanning speed can be relaxed. This allows the use of a simple mechanical scanning mechanism to sweep a laser sheet across a large volume of interest in order to capture the asymmetric structure of a flame confined within an annular combustion chamber. The use of a LIF-based method allows a single flame in the annular rig to be highlighted and measured despite the presence of neighbouring flames; an undertaking that would be difficult using a CTC-based approach.

The remainder of the paper is organised as follows: Sect. 2 outlines the annular configuration and the details of the experimental technique. The technique is also evaluated in Sects. 3 and 4, using a numerical experiment that produces synthetic PLIF images of an idealised object, in order to estimate error associated with the experimental and reconstruction parameters. The experimental reconstruction of a flame in an annular rig is then presented Sect. 5.

## Experimental methods

### Annular combustor

The annular combustor which was reported previously by Mazur et al. (2019) features 12 equally spaced turbulent bluff body stabilised flames. The main features and dimensions will be recalled here briefly. An ethylene/air mixture was introduced through a cylindrical plenum chamber of diameter, $D_p=212$ mm. Two mesh screens were located at the plenum inlet and outlet to improve flow uniformity. The flow is divided around a hemispherical body with a diameter of $D_h=140$ mm, into 12 injection tubes of length $l_i=150$ mm, each with a diameter $D=18.9$ mm.

The injectors were equally spaced around a diameter of $D_{inj}=169$ mm, and each was fitted with a $D_b=13$ mm diameter bluff body flush mounted with the annular chamber at the location referred to as the dump plane. The bluff bodies are centrally mounted on a rod of diameter $D_r=5$ mm, and have a half angle of $45^\circ$ and a blockage ratio of approximately 50 %. Each bluff body is centered using three grub screws, which are located $L_g=50$ mm upstream of the dump plane. The annular enclosure consisted of a stainless steel inner wall with a diameter of $D_i=127$ mm and an outer cylindrical quartz wall to permit optical access with a diameter of $D_o=212$ mm.

The reactant mixture was maintained at an equivalence ratio of $\phi =0.7$ and a bulk velocity of $U_b=17$ m/s at the dump plane. The equivalence ratio and flow velocity were chosen as these correspond to a thermoacoustically stable operating condition (Mazur et al. 2019). Dynamic pressure measurements were conducted using six pressure sensors at upstream locations inside three of the injector tubes, to confirm that the operating point remained thermoacoustically stable. The combustor was operated for approximately 20 seconds prior to data acquisition, in order to reach a quasi steady thermal state and maintain consistency between experiments.

### Scanning OH-PLIF

OH radicals were excited using a Sirah Credo dye laser, pumped by a Nd:YAG Edgewave IS 400-2-L laser with a pulse energy of 10 mJ at 10 kHz. The laser wave length was regularly tuned to maximise the OH signal at approximately 283.54 nm with a dye laser power of $\sim$ 0.25 mJ per pulse. A series of optics were used to produce a laser sheet with a thickness with a mean value of $\delta \approx 0.4$ mm. The laser sheet thickness was measured using calibration images, as described in Sect. 2.3.

A Galvanometric scanning dynAXIS XS UV coated mirror is used to sweep the UV laser sheet across the flame, over a total depth of 40 mm. The position of the mirror was controlled using a voltage signal with a range of 0.5 V and precision of $4\times 10^{-5}$ V, which results in a sheet positional precision of 0.003 mm. A lens is not used to realign the sheet angle following the galvanometer, as the sheet is also passed through the curved surface of the outer quartz combustion chamber wall, resulting in an array of incidence angles due to refraction. Instead the location of each laser sheet is defined through calibration, as described later in Sect. 2.3. A total of 200 laser sheet positions were used, which based on the measured sheet thickness of $\delta \approx 0.4$ mm, results in a sheet overlap of 50%.

**Fig. 1:**
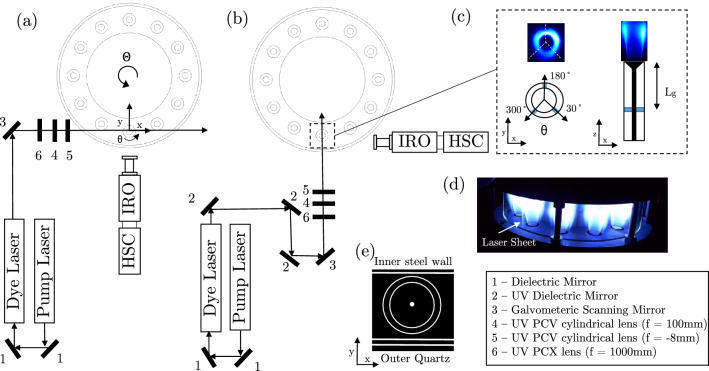
Schematic of experimental setup viewed from above for **a** Scan XZ and **b** Scan YZ, **c** representation of injector from top and side views with positioning of grub screws in blue and images of OH chemiluminescence, **d** Image of annular rig during experiment **e** orientation of inner and outer annular walls

Two Phantom V2012 cameras equipped with Lavision Intensified Relay Optics and 100 mm focal length Cerco 2178 UV lenses were used to conduct the OH-PLIF measurements. The first camera equipped with a 310 nm UV filter (Full Width Half Maximum(FWHM) = 10 nm) imaged from the side and collected OH-PLIF measurements, while the second camera imaged from overhead, via an air cooled mirror, and was used to conduct the laser sheet calibration. Both cameras equipped with the 310 nm UV filters were additionally used to collect OH$^*$ chemiluminescence measurements of the flame. Side view OH$^*$ images were collected while all 12 flames were on using the camera arrangement shown in Fig. 1a in order to avoid capturing intensity from neighbouring flames. The side view resulted in a pixel resolution of $800\times 896$ pixels, resulting in a spatial resolution of 10 pixels/mm for the PLIF measurements. The overhead camera used a resolution of $896 \times 800$ pixels to image an area of 40 mm $\times$ 40 mm, providing a resolution of 10 pixels/mm. Both PLIF and OH$^*$ measurements were conducted at an imaging frequency of 10 kHz. An aperture setting of f/8 was used for the PLIF measurements to ensure a sufficient depth of field. For the OH-PLIF measurements, at each sheet location a total of 354 images were acquired, resulting in a total of 70800 images for each scan. Four independent scans were performed to overcome the limitations of the camera’s buffer size, resulting in a total of 283200 images.

A bias error was encountered when performing the Scanning-PLIF measurements (which is discussed in detail in Sect. 4), and therefore Scanning-PLIF measurements were made using two perpendicular scan directions, as shown in Fig. 1. These are labelled and referred to in the following as Scan XZ and Scan YZ, which correspond to the predominant orientations of the laser sheet and imaging planes. Care was also taken to ensure the laser sheet thickness and image spatial resolution were kept relatively constant for the two scan directions. Calibration is conducted to a common coordinate system, allowing the reconstructions to be combined, which is described in the next section.

### Camera and laser sheet calibration

In order to reconstruct the 3D flame structure, two calibrations were employed: First a camera calibration model was used to define each pixel’s line of sight through the volume, for both overhead and side views; and secondly a laser sheet calibration was used to define the spatial location and orientation of each each laser sheet in the scan. Calibrations of the two cameras and different scan directions were aligned with a single global coordinate system through the identification of a common reference point (center of the bluff bodies) and vector (orientation angle of the calibration plate).

The side camera calibration was performed by imaging a calibration target which was traversed in increments of 3 mm through a depth of 42 mm, resulting in 15 planes of calibration data. A third order polynomial model was used for the image to real space transformation, in order to account for refractive index effects of the curved quartz enclosure. The calibration of the side camera from both scan directions produced a mean error of 0.45 pixels (0.046 mm) with respect to all planes, and the overhead calibration produced a mean error of 0.08 pixels (0.008 mm). During the side camera calibration the plate was carefully aligned so that its face was perpendicular to the dump plane. Images of the calibration plate were taken using the overhead camera during the traverse. These images allow the plate position and therefore lines of sight from the side camera to be related to a global coordinate system in the frame of reference of the annular combustor.

In terms of the laser sheet calibration, different laser sheet locations were recorded by synchronising the camera signal and the signal sent to the scanner to move the laser sheets. First the vertical orientation of the laser sheets was assessed by positioning an angled calibration target in the volume, imaged from the side camera. Next, the orientation of the laser sheet on the dump plane was assessed. As the reflection of laser light from the dump plane of the annular combustor is weak, a reflective target was placed on the dump plane to ensure that the laser sheet was visible to the overhead camera. A total of 100 overhead images were averaged for each laser sheet position. The mean sheet location on the dump plane and the vertical orientation were calculated to sub-pixel accuracy from the two views, and a Gaussian distribution was fitted to characterise the beam width in terms of an intensity profile. The sheet coordinates were converted into real space through the overhead camera calibration.

The real space coordinates of all laser sheets were then fitted to a single laser sheet calibration model using a least squares approach. An equation of plane could then be defined for each laser sheet location from the calibration model.

The intersection of each pixel’s line of sight from the side camera, as defined by the camera model, with the real space coordinates of the laser sheet plane can be used to project images into real space during the volume reconstruction. This process was done for both scanning directions and the definition of the global coordinate system allowed for alignment of the reconstruction from the two different scan directions.

### Volumetric flame surface density

The PLIF images were used together with the laser and camera calibration models to calculate the 3D distribution of the Flame Surface Density (FSD). The FSD is defined as the flamelet surface area per unit volume under, averaged over time, the assumption of flamelet framework for premixed turbulent flames (Zhang et al. 2015). FSD will be used later in Sect. 5.4 to describe the flame structure and localised heat release rate.

FSD has been used previously in premixed flames as an indicator of heat release rate (Paul and Najm 1998; Knikker et al. 2002; Dawson and Worth 2014). A study by Balachandran et al. (2005) calculated the 2D Flame Surface Area (FSA), which represents the flame length per unit area, from a single measurement plane and inferred a 3D distribution of FSD by rotating this under the assumption of axisymmetry. The resulting integrated FSD was then related to integrated $OH^*$ measurements of heat release rate fluctuations, showing good agreement. However, the authors highlight that FSD calculated in this manner neglects fine scale wrinkles typically present along the surface of turbulent flames. It also by construction neglects out-of-plane wrinkling, a detailed discussion of this limitation is included in Sect. 4.

In a similar manner, this study will relate the FSD determined from the reconstructed volumetric distribution of flame surface to the volumetric heat release rate, neglecting flame surface wrinkling beyond the measurement resolution, but preserving out-of-plane variations. While the 2D calculation of FSA uses edge locations binned into pixels, the 3D calculation of FSD relies on edge locations reprojected into a 3D volume, and binned into 3D voxels. This approach can be considered the 3D analog of 2D FSA calculations (Veynante et al. 1994; Zhang et al. 2014; Balachandran et al. 2005).

To determine instantaneous flame front locations PLIF images were first processed to correct for non-uniformities in the beam profile, and to accentuate the division between products and reactants. An intensity correction was applied to each image in both the horizontal and vertical directions which corrects for the Gaussian shape of the expanded laser sheet, and absorption as the beam passes through the combustor.

**Fig. 2:**
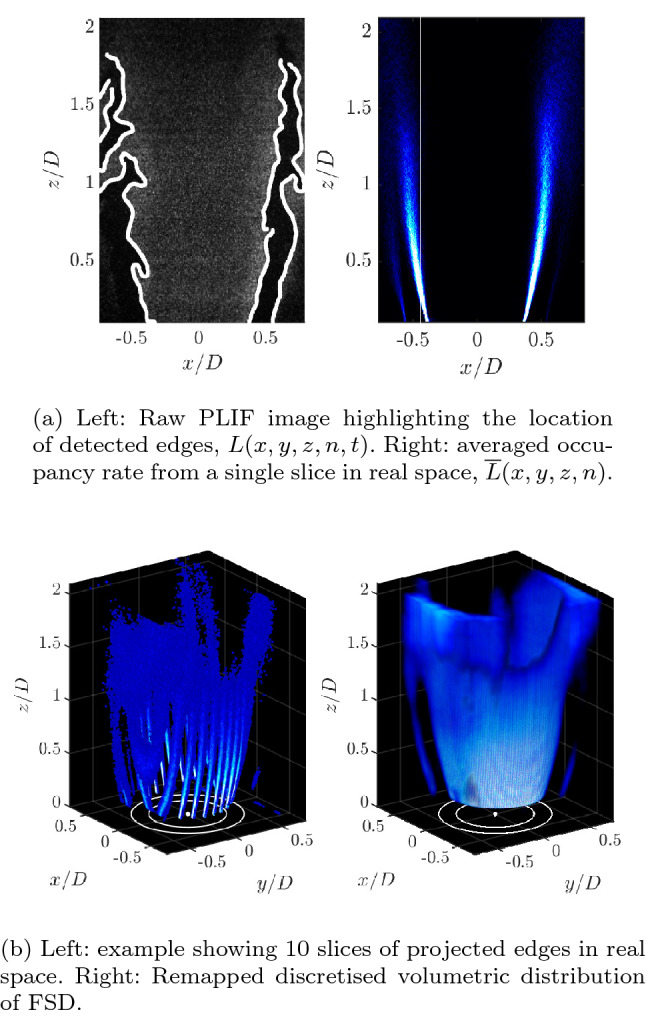
Example calculation of volumetric Flame Surface Density

**Fig. 3:**
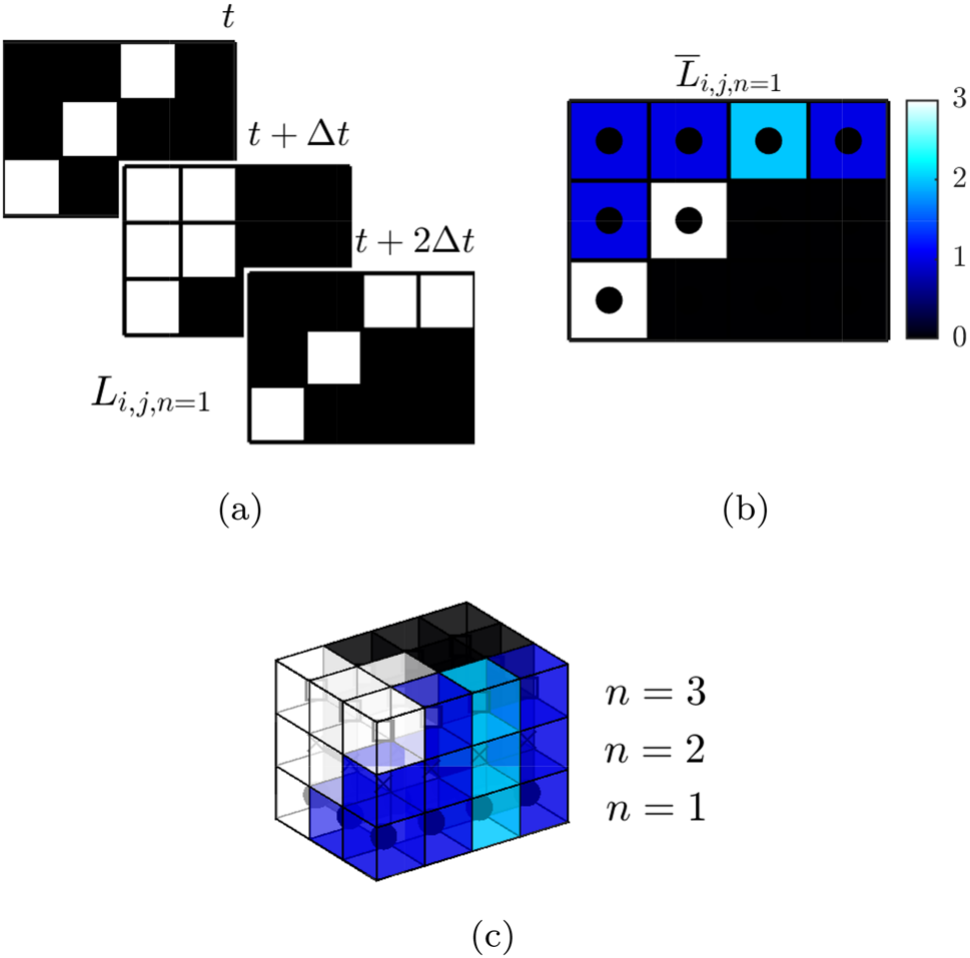
Illustration of FSD calculation: **a** time averaging of flame edges, **b** time averaged flame edge for sheet number 1, dots represent center of pixel **c** Three-dimensional view of multiple sheets in real space prior to FSD calculation

An edge preserving Gaussian filter and a nonlinear diffusion filter were then applied to the images to remove noise, before a Canny edge detection algorithm is applied to find the edge contours of the flame front separating burnt and unburnt gas. A gradient threshold was also applied during the edge detection procedure to remove any non-physical artefacts that were the result of intensity inhomogenity. An example of the raw PLIF image and the identified flame edges is shown in Fig. 2. Edge detection results in a sequence of instantaneous binary images, *L*(*i*, *j*, *n*, *t*), in which the flame front location is defined by pixels with a value of 1, for each laser sheet location, *n*, in image space (*i*, *j*). A schematic example of this process is shown in Fig. 3a using three binary images representing flame edges at three instants in time.

It is important in the later reprojection step to ensure that the edge point count is not biased towards any of the three sampling directions (two in the imaging plane, and one in the laser sheet scanning direction). This is accomplished by binning the flame edge images such that the final image resolution matches the sheet spacing of 0.2 mm, preventing biasing of the FSD calculation in the laser scan direction. The laser sheet orientation is very close to orthogonal with the voxel layers in the discretised volume, meaning a correction is not required to account for the divergence of adjacent laser sheets in different parts of the volume.

The probability of flame front location or occupancy rate, ${\overline{L}}(i,j,n)$, is calculated by time-averaging all binary images from a single laser sheet location, *n*. Each pixel in ${\overline{L}}(i,j,n)$ was then projected onto the global coordinate system using the laser sheet and camera calibration models, to calculate the real space distribution of the time-averaged occupancy rate, ${\overline{L}}(x,y,z,n)$, for each laser sheet location, *n*. The result of the projection operation can be seen for a number of example planes from a single scan direction in Fig. 2. Again to illustrate the process schematically, an example of this calculation is shown in Fig. 3b, with the occupancy rate calculated from the three binary images in Fig. 3a.

To create a regular array of data, a volume was discretised at a resolution of 5 voxels/mm which was chosen to match the sheet displacement resolution in the scan direction, and the scattered data, ${\overline{L}}(x,y,z,n)$, was remapped onto this volume using a scattered linear interpolation scheme. This volumetric distribution of edge locations represents the time-averaged flame surface area, $\overline{A_f}(x,y,z)$. Again a visual representation of multiple sheets and respective voxels with ${\overline{L}}(x,y,z,n)$ are shown schematically in Fig. 3c as an example of this process. The FSD can then be calculated using Eq. 1  (Zhang et al. 2014),

1 $$\begin{aligned} \mathrm{FSD} = \lim _{\Delta x\rightarrow \ 0} \frac{\langle {\overline{A}}_f \rangle }{\Delta x^3} \end{aligned}$$

where $\langle {\overline{A}}_f \rangle$ is the time-averaged surface area of flamelets, spatially integrated within a $5\times 5\times 5$ voxel cubic interrogation volume, $\varDelta x$. The interrogation volume dimensions were chosen based on the findings of Donbar et al. (2000), and is smaller than the flame brush width. The FSD at each voxel location is therefore determined by the sum of ${\overline{A}}_f$ over the interrogation volume centred at that voxel, normalised by the interrogation volume. The volumetric FSD distribution of the experiment are shown in Fig. 2. The FSD is approximately proportional to the time-averaged distribution of the heat release rate, given that the heat release rate per flame surface area is relatively constant for fuel-air mixtures with unity Lewis number and are in the wrinkled flamelet regime, $Q(x,y,z)\approx \mathrm{FSD}(x,y,z)$.

It is useful to examine the local azimuthal variation of the heat release rate around the flame, and therefore the domain can be recast in cylindrical polar coordinates (as in Fig. 1c), and wedge shaped regions investigated. Each wedge, $Q_\theta$, corresponds to the integrated FSD within a range of local azimuthal angles and downstream distances, as defined by Eq. 2.

2 $$\begin{aligned} Q_{\theta , z} = {\sum \limits _{r}\sum \limits _{z=z_1}^{z_2} \sum \limits _{\theta =\theta _1}^{\theta _2} \mathrm {FSD}(r,\theta ,z)} \end{aligned}$$

Here, a centre wedge angle, $\theta _c$, and angular wedge extent, $\theta _w$, are defined to produce the two azimuthal limits, $\theta _{1,2}=\theta _c\mp \theta _w$, and $z_1$ and $z_2$ are the longitudinal limits. A wedge angle of $\theta _w=10^\circ$ is employed in the present study.

## Synthetic experiment method

Despite previous implementations of Scanning LIF, the influence of experimental parameters have not been fully described. Therefore, in the current study, synthetic PLIF images were generated and processed in order to optimise the setup, and also to better understand any inherent weaknesses of the approach. The use of a synthetic experiment allows an ideal synthetic flame object of known geometry to be defined, enabling quantification of the errors associated with the reconstruction procedure, and the experimental parameters. Specifically, the synthetic experiment was used to understand the error associated with the laser sheet thickness, spacing, and orientation as well as image noise. Tests were also conducted to understand the ability of the method to resolve contorted flame fronts.

### Synthetic PLIF image generation

**Fig. 4:**
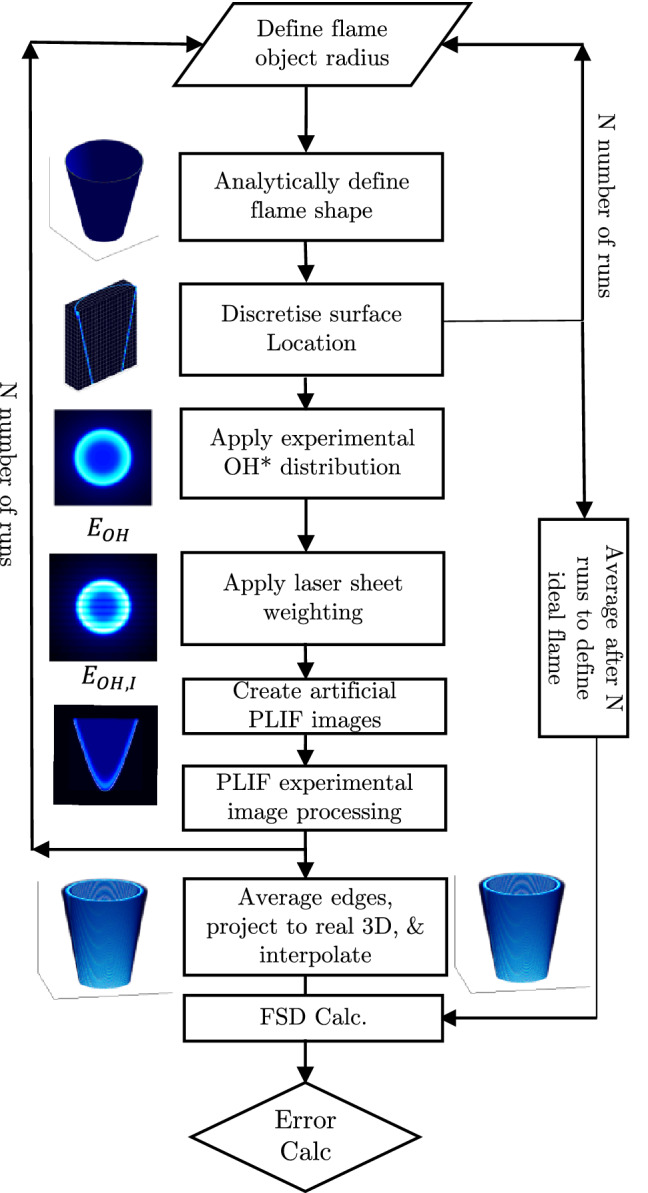
Synthetic PLIF image generation and processing flow diagram

Figure 4 shows a flow diagram of the synthetic experiment process, which will now be described. In order to generate synthetic PLIF images, an ideal flame surface object is first defined, which describes the exact location of a surface. For simplicity a conic section was used to define the surface position, as this is geometrically similar to a bluff body stabilised V-flame. The cone surface is defined by $r^2 = (5.7z + D_b/2)^2$ over a domain size of $40 \times 40 \times 40$ mm$^3$. The present approach is aimed at reconstructing the time-averaged flame shape. Therefore, the radius of the ideal flame object was varied to replicate the unsteady flame motion. Flame objects were created with radii following a probability density function determined from the experimentally measured flame brush. In practice the variation of object radius is Gaussian in form with a standard deviation of 1.3 mm. A total of 160 instantaneous snapshots are used to simulate each time-averaged condition.

To replicate flame front curvature effects, sinusoidal variations of varying amplitude and frequency were added to the conic section surface. The amplitude and frequency were selected in order to replicate similar levels of curvature measured directly from the extracted flame edges. However, adding realistic levels of flame front curvature did not significantly affect the reconstructions, and therefore for brevity, these results are not reported herein.

Surface points on each flame object were first determined from the cone surface equation and then discretised onto a 3D array of voxels by using a scattered linear three-dimensional interpolation scheme. The volume was discretised at 5 voxels/mm which is consistent with the experiment. The average of all discretised flame objects is described as the ideal flame object surface, ${\overline{A}}_{f,ideal}$, and flame surface density $\mathrm{FSD}_{ideal}$.

A representative experimental flame front intensity profile was determined directly from the detected flame front edges captured by the experimental measurements. This is described in detail in Appendix 1.

The distribution is applied normal to the flame surface location in order to produce a discretised ideal three-dimensional intensity distribution of OH. For convenience this operation is performed directly on the discretised flame object. The flame surface normal at each nonzero voxel in this discretised object is defined based on the equation of the cone, and intensity is distributed along this normal, scaled by the original voxel intensity, to produce a discretised flame object OH distribution, $E_\text {OH}$. The resulting intensity volume, $E_\text {OH}$, is then used to produce artificial PLIF images.

To replicate the fluorescence of OH, the laser illumination was modelled with a Gaussian intensity profile. An equation of plane was used to define the laser sheet location, and the illumination intensity in the volume was varied according to the normal distance from the plane, using Eq. (3) similar to that represented by (Lawson and Dawson 2014).

3 $$\begin{aligned} I(\mathbf{X })=\exp (-8(d)^2/\delta ^2) \end{aligned}$$

Here $\mathbf{X }$, defines the coordinates of a plane, *d* represents the normal distance from the plane, and $\delta$, represents the sheet thickness. In this synthetic study, the laser sheet normal is oriented in the *y* direction. The resulting distribution was used to calculate the illumination in each voxel of the discretised volume, $E_\text {I}$. The illumination volume was then used to scale the intensity in $E_\text {OH}$ for each sheet location to produce $E_\text {OH,I}=E_\text {OH}\cdot E_\text {I}$. For simplicity, laser sheet inhomogeneity and laser absorption across the field of view were neglected as these effects are removed somewhat through intensity correction when post processing PLIF images.

Synthetic PLIF images were generated by projecting each nonzero voxel of the volume, $E_\text {OH,I}$, onto the image plane, using a simple pinhole camera model, with a similar spatial resolution as the experimental setup. The use of an ideal camera model eliminates potential error from camera calibration, although this can be taken into account through purposeful misalignment during parametric testing. It should be noted that while these synthetic images are not fully representative of the actual experimental images, they are sufficient for optimising the processing procedure, and provide insight into potential sources of error.

Synthetic images were produced for each laser sheet location, *n*, and were passed through the edge detection algorithm, to evaluate the simulation edge occupancy rate, $L_{sim}(i,j,n,N)$, in the image plane for each flame object snapshot, *N*. After each loop, the radius was updated and a new snapshot was processed. Averaging all snapshots allowed the average edge occupancy rate ${\overline{L}}_{sim}(i,j,n)$ to be calculated. The same process that was applied to the experimental results is then used to reconstruct the 3D discretised volumetric representation of the flame surface density, $\mathrm {FSD}_{sim}(x,y,z)$. This can then be compared to an ideal flame object flame surface density, $\mathrm {FSD}_{ideal}$, as described in Sect. 3.2.

### Error estimation

Defining the ideal flame surface with an exact expression allows for a direct assessment of reconstruction accuracy, which is quantified through the normalised correlation coefficient *R* in Eq. (5), and is similar to that defined by (Elsinga et al. 2006).

4 $$\begin{aligned} R = \frac{\sum _{x,y,z} \mathrm{FSD}_\text {ideal}(x,y,z) \cdot \mathrm{FSD}_\text {sim}(x,y,z)}{ \sqrt{\sum _{x,y,z} (\mathrm{FSD}_\text {ideal}(x,y,z))^2 \cdot \sum _{x,y,z} (\mathrm{FSD}_\text {sim}(x,y,z))^2}} \end{aligned}$$

The combination of a cylindrical flame object with a single scanning direction introduces asymmetry, and it is also of interest to consider the local reconstruction quality. The domain can be recast in cylindrical polar coordinates, and the reconstruction quality of wedge shaped regions can be investigated by applying Eq. 4. Again a wedge angle of $\theta _w=10^\circ$ is employed.

5 $$\begin{aligned} R_\theta = \frac{\sum \nolimits _{r,z}\sum \nolimits _{\theta =\theta _1}^{\theta _2} \mathrm{FSD}_\text {ideal}(r,\theta ,z) \cdot \mathrm{FSD}_\text {sim}(r,\theta ,z)}{ \sqrt{\sum \nolimits _{r,z}\sum \nolimits _{\theta =\theta _1}^{\theta _2} \mathrm{FSD}_\text {ideal}(r,\theta ,z)^2 \cdot \sum \nolimits _{r,z}\sum \nolimits _{\theta =\theta _1}^{\theta _2} \mathrm{FSD}_\text {sim}(r,\theta ,z)^2}} \end{aligned}$$

## Synthetic experiment results

A series of parametric studies were conducted to help understand the accuracy of the method to changes in laser sheet thickness, sheet overlap, and image noise.

**Fig. 5:**
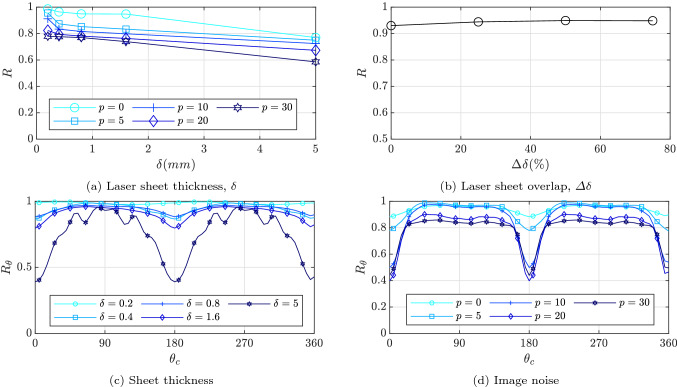
Global reconstruction quality as a function of laser sheet thickness and overlap **a** and **b**. Default values of $\delta =0.4$ mm, $\varDelta \delta =0\%$ (zero percent overlap represents sheets that are adjacent to each other), and zero image noise were used. Local reconstruction quality as a function of azimuthal location with respect to **c** sheet thickness with 50 percent overlap and **d** image noise with $\delta =0.4$ mm and $\varDelta \delta =50\%$

The sensitivity of reconstruction quality due to varying sheet thicknesses and noise are presented in Fig. 5a. A fixed sheet overlap of $\varDelta \delta = 0\%$ is applied, and spatially white, non zero mean and random noise is added with levels ranging from $p=0\%$ to 30%. The noise level is with respect to the maximum intensity of each image. For thin sheets the reconstruction accuracy is high, and as sheet thickness increases, the reconstruction accuracy decreases slowly. Thin sheets capture images with sharp OH gradients, improving the edge finding, and leading to higher reconstruction accuracy. In the experiment the laser sheet thickness is controlled by arrangement of the sheet optics, and cannot be arbitrarily thin. However, the thickness of $\delta = 0.4$ mm yields high correlation values, giving confidence that this is suitable. The addition of noise also reduces the reconstruction accuracy, which is expected since it decreases the precision of the edge finding algorithm. It should be noted that while noise levels up to $p= 30\%$ were tested, these may be considered extreme, and typically experimental noise is around $p \approx 10\%$. Even a modest addition of noise at this typical level results in a significant reduction in reconstruction correlation, which is discussed further below.

The sheet location is controlled in the experiment through the galvanometer mirror alignment, allowing an almost arbitrary choice of sheet overlap, $\varDelta \delta$. Increasing the overlap increases the amount of data available, resulting in a potential improvement in resolution in the scan direction. However, when sheets overlap, the measurements are no longer necessarily independent. The reconstruction sensitivity to overlap was investigated based on a sheet width of $\delta = 0.4$ mm, with zero image noise. While the combination of overlap and sheet width define the number of sheets required to illuminate the domain, $E_{OH}$, for nonzero overlap values, a total of 100 sheets were used to illuminate the $\varDelta \delta =0$ case. Figure 5b shows that the reconstruction accuracy increases marginally with increasing overlap, reaching a maximum at $\varDelta \delta \approx 50\%$. Therefore, while high overlap is desirable, the greater the overlap, the greater number of laser sheet locations will be required to illuminate a volume of interest, requiring a greater amount of data to be stored and processed. Based on these simulations an overlap of $\varDelta \delta = 50\%$ was chosen in the current experiment.

The use of a rotationally symmetric flame object allows the local azimuthal reconstruction accuracy, $R_\theta$, to be assessed in order to further demonstrate the location of the reconstruction error, as shown in Fig. 5c. A clear angular dependence on reconstruction accuracy can be observed. The highest correlation values are seen at angles of $\theta _c=90^\circ$, $270^\circ$, where the laser sheet is almost perpendicular to the flame surface. The reconstruction accuracy is shown to decrease with orientation angle, reaching a minimum for $\theta _c=0^\circ$ and $180^\circ$, at which angles the laser sheet normal and flame surface normals are close to parallel. The local reconstruction accuracy also depends on the laser sheet width, with larger reductions in accuracy observed at $\theta _c \approx 0^\circ$ for larger laser sheet widths in comparison with $\theta _c \approx 90^\circ$.

This angular dependence of the reconstruction accuracy is an important source of bias error in the current method, which can be explained through the change in OH gradients on synthetic PLIF images. When laser sheets are orthogonal to the flame surface, the synthetic PLIF images have high OH gradients, which can be easily identified using the edge detection algorithm. However, when the laser sheet and flame surface alignment is closer to parallel, due to the finite laser sheet thickness, the OH gradient becomes much shallower. Shallower OH gradients result in larger uncertainty in the edge detection stage, resulting in lower reconstruction accuracies. Similarly, increasing the laser sheet thickness also reduces the magnitude of the OH gradient, resulting in reduced accuracy.

The local azimuthal reconstruction was also evaluated for simulations of different noise levels. A sheet thickness of $\delta = 0.4$ mm with an overlap of $\varDelta \delta = 50\%$ was used, and the noise sensitivity is presented in terms of the local reconstruction accuracy in Fig. 5d. The addition of noise reduces the reconstruction accuracy. However, the greatest decrease in accuracy occurs when the laser sheet and flame surface are close to parallel, $\theta _c \approx 0^\circ$ and $180^\circ$. The effect of noise compounds the shallow OH gradients resulting in large positional errors in the edge finding.

**Fig. 6:**
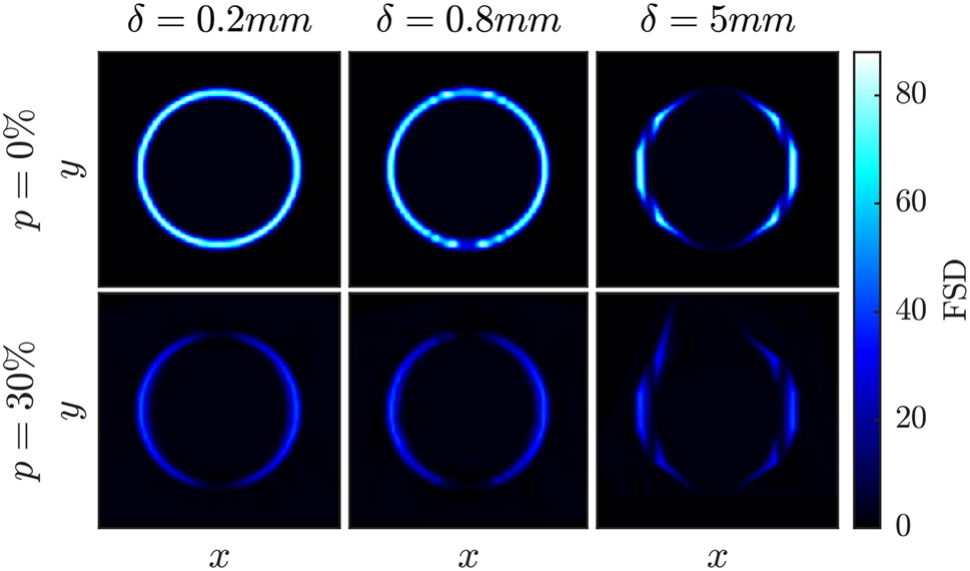
Simulation result of $x-y$ slice at $z = 20$ mm, representing the reconstructed flame object, $\mathrm{FSD}_{sim}$ with varying sheet thickness, $\delta$, and image noise, *p*

To better understand the decrease in accuracy observed, Fig. 6 shows a visual representation of a single slice of the $\mathrm {FSD}_{sim}$ volume in the $x-y$ plane for selected laser sheet thicknesses and noise levels. When the laser sheet is thin ($\delta = 0.2$ mm) and for zero image noise ($p = 0\%$) the reconstruction is symmetric and has uniform intensity. However, when the laser sheet thickness is increased the upper and lower edges of the reconstructed object lose intensity, and for large sheet thicknesses the circular shape becomes distorted. The detected edges show larger spatial deviations, and in some cases the edge detection fails completely and edges are not captured at all. This bias is further amplified by the addition of noise which reduces the ability of the edge detection algorithm to find edges at the upper and lower parts of the object shown in Fig. 6. At these locations the laser sheet orientation is tangent to the flame surface. Therefore, while Fig 5c does not show any bias errors for very thin laser sheets ($\delta =0.2$ mm), bias is always observed in the presence of noise.

The synthetic Scanning PLIF experiments demonstrate the reasonable accuracy that can be expected based on the obtainable experimental laser sheet thickness and an overlap of 50%, even for reasonably low signal to noise ratios. However, the numerical study also demonstrated a clear bias associated with the orientation of the laser sheet and flame surface. While high values of correlation coefficient are shown for thin sheets and low noise levels, positional biases in finding edges close to flame fronts may never be completely eliminated, which is discussed further in Appendix 2. It is worth highlighting that such a bias will not just affect the present measurements, but given the highly turbulent nature of many flows of interest, such errors may be present but not reported in a wide range of studies. To mitigate this bias error it was therefore decided to employ two almost perpendicular scan directions as described previously.

## Experimental results

### OH* chemiluminescence

**Fig. 7:**
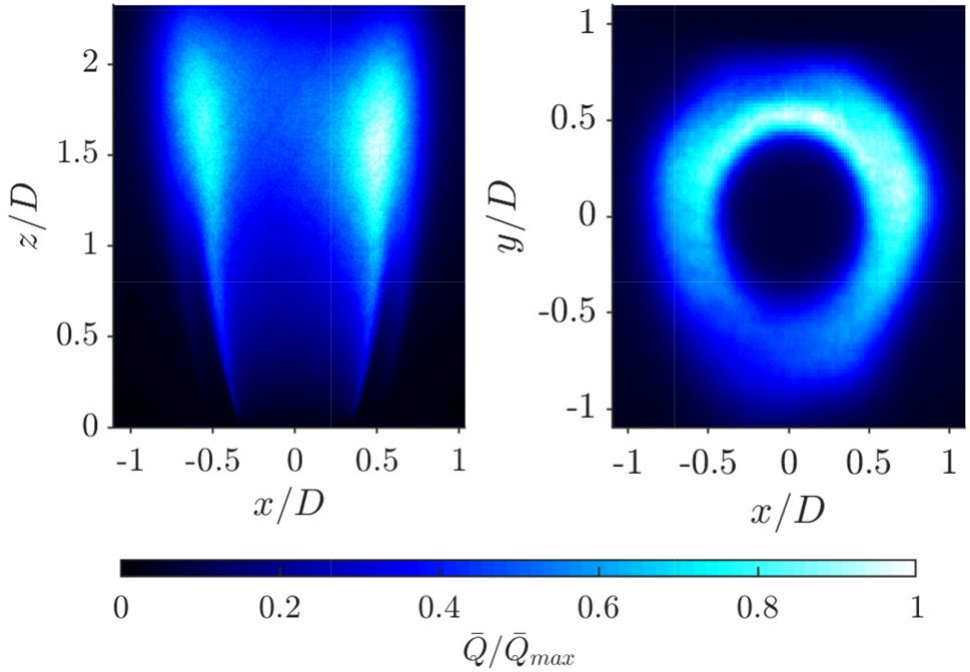
Normalised averaged OH chemiluminescence of 10000 time resolved images from side view of Scan XZ (left) and top view (right). Colour bar represents the time-averaged mean intensity, $\bar{Q}$ normalised by its maximum, $\bar{Q}_{max}$

Before presenting the OH PLIF results, it is useful to describe the flame using a conventional integrated line of sight method. OH$^*$ images were captured of the flame from the $x-z$ plane and overhead, and are shown in Fig. 7. The flame structure is similar to that observed previously (Mazur et al. 2019), with the flame stabilised primarily along the inner shear layer separating the annular jet and the recirculation zone above the bluff body. Some flame elements are also observed close to the flame tip along the outer shear layer. Despite the nominally axisymmetric inlet conditions, the flame shows significant asymmetry. This can be observed in the side view, with higher intensity observed on the right hand side of the flame. The overhead view also shows clear asymmetry between regions close to the inner and outer annulus.

The asymmetry has a number of potential sources: First, the bluff body is centred with three grub screws which is known to introduce azimuthal asymmetry around the flame (Æsøy et al. 2020); second, the flame is confined in annular geometry, with a quartz outer annulus, a metal inner annulus, and neighbouring flames either side; third, the bluff body is expected to be imperfectly centered, and small manufacturing asymmetries may also be present; and forth, the flow from the cylindrical plenum upstream may not be perfectly uniform, due to the relatively short inlet tube length, $l_i$. From above, the OH$^*$ distribution has a slightly triangular shape, which is perhaps a result of the three grub screws. The region closest to inner wall has a higher intensity and appears wider in comparison with the region close to the outer wall, which is likely a result of the differing thermal conductivities associated with quartz and metal enclosure walls, and potentially the curvature of these. While it is difficult to fully describe the asymmetry based on integrated line of sight methods, this asymmetry will be described further through the volumetric PLIF measurements presented in the next section.

### Instantaneous OH PLIF measurements

**Fig. 8:**
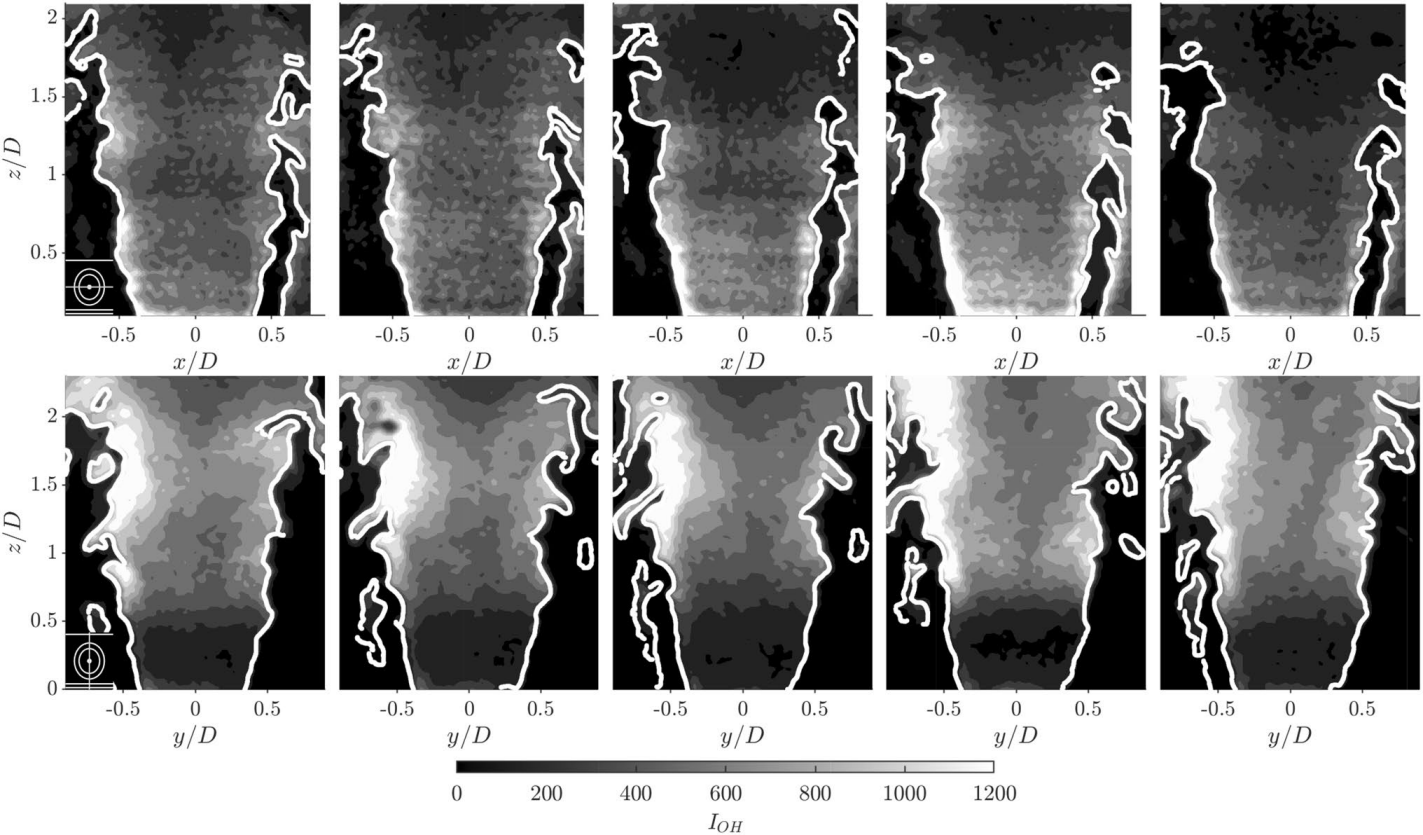
Time Resolved OH PLIF images from two scan directions, along burner centre. Raw PLIF images were preprocessed using a Gaussian filter. Scan XZ (top row), Scan YZ (bottom). $\varDelta t$ of 0.2 ms between each image. Colour bar represents, $I_{OH}$, spatial distribution of OH fluorescence

The time-dependent behaviour of the flame can be directly assessed using instantaneous snapshots obtained from the high speed OH-PLIF measurements. Processed PLIF images are shown in both scan directions in Fig. 8, with the laser sheet in both instances passing through the bluff body centre. A small schematic representation of the burner showing the laser sheet orientation is included in the lower left corner of each time series. The PLIF images clearly show a cross section of the flame, cutting through the annular jet in two locations. However, it is interesting to compare these two cross plane slices.

The top row images were taken when the laser sheet was approximately aligned with the $x-z$ plane, and therefore this plane highlights the influence of flame-flame interactions between adjacent flames in the annulus. The $y-z$ images in Fig. 8 highlight the flame interaction with the annular walls. The flame fronts for both planes are highly contorted, due to the turbulent nature of the flow. Large scale flame wrinkles can be observed forming in the flame sheet as the flow moves downstream, resulting in flame sheet fragmentation in the upper part of the domain. In comparison with the bottom row, the flame slices in the top row exhibit a larger number of flame elements stabilised along the outer shear layer. The presence of a neighbouring flame is likely to increase the outer recirculation zone temperature, resulting in a higher probability of stabilising the flame on the outer shear layer. The presence of flame elements in the outer shear layer permits more flame channel closing events to occur, where flame fronts stabilised on inner and outer shear layers propagate into each other resulting in the formation of pockets of unburnt gas Worth and Dawson (2019). The events can be identified on the right hand side of flame in the top row, and unburnt gas pockets are observed to pinch-off and then be advected downstream.

### Planar distributions of flame occupancy rate

**Fig. 9:**
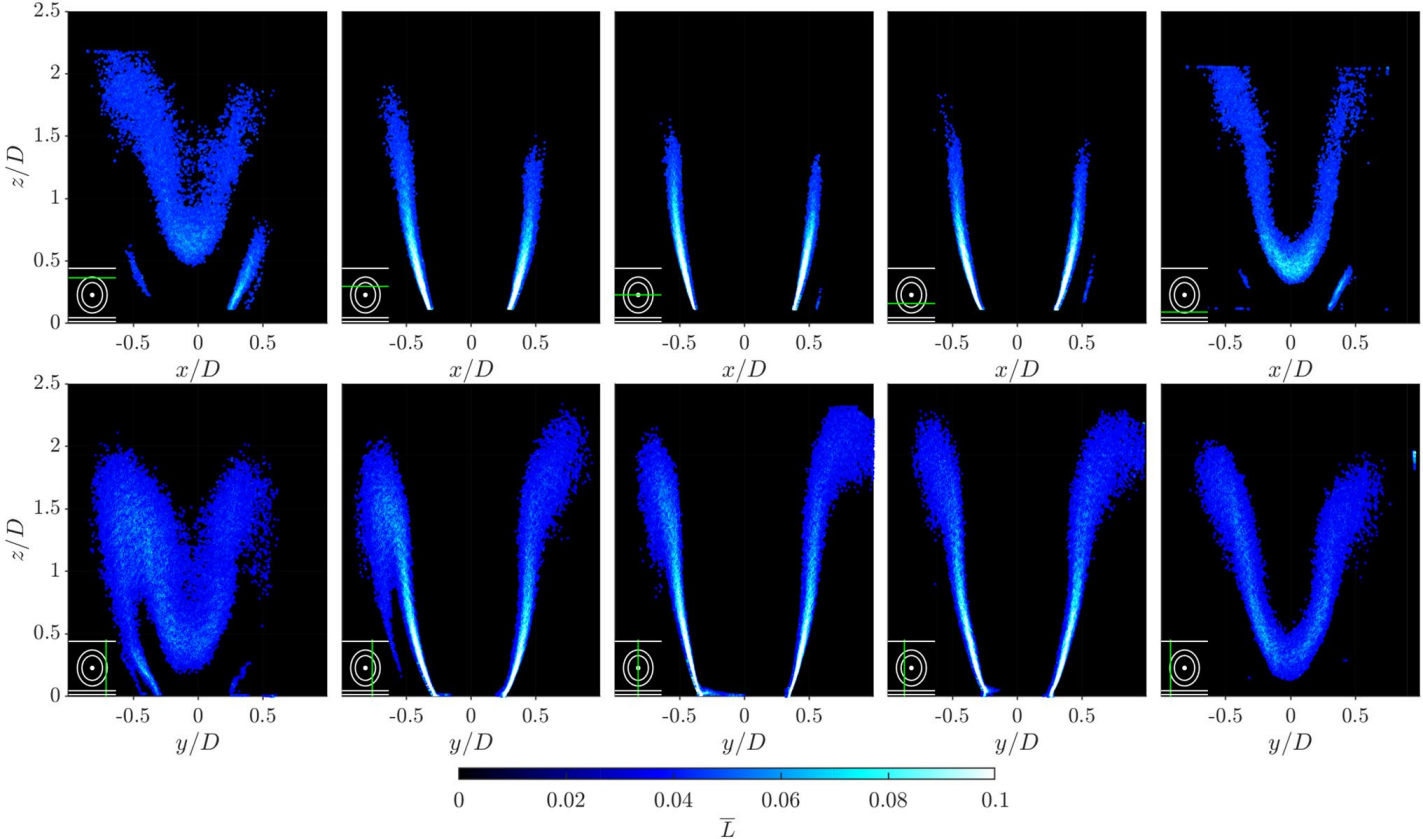
Slices of projected averaged flame occupancy rate, ${\overline{L}}$, in Scan XZ (top row) and Scan YZ (bottom row). Slice position represented by green line relative to the bluff body from overhead view of the burner, laser positions are at -0.48D, -0.24D, 0D, 0.24D, 0.48D in the direction of each scan

Before considering the reconstructed volumetric distribution, it is useful to examine the mean projected occupancy rate, ${\overline{L}}$, as way to better understand the previously observed asymmetries. Figure 9 shows the distribution of ${\overline{L}}$ for both scan directions, at cross section locations through the flame. To help identify the laser sheet orientation, again a small schematic representation of the burner is included in the lower left corner of each sub figure.

Comparing first the ${\overline{L}}$ distributions when the laser sheet passes through the burner centre (middle column), the change in flame height can be clearly seen. In the $x-z$ plane in the upper row, the turbulent flame brush reaches a maximum height of $z/D \approx 1.5$, whereas in the $y-z$ plane it extends downstream much further to $z/D \approx 2.2$. Asymmetries are also observed between left and right hand sides of the flame in both scan directions. In the $x-z$ plane in the upper row, the slightly higher distribution on the left hand side is likely to result from imperfections in the bluff body alignment. However, a much larger asymmetry is observed in the $y-z$ plane in the bottom row, with a higher and wider flame brush that interacts with the inner annular wall on the right hand side, in comparison with the shorter more compact flame brush on the left hand side.

The source of this asymmetry may be due to either the effective confinement of the flame, or the material properties of the inner and outer walls. Due to the annular enclosure, the flow has a greater area to expand into towards the outer annular wall in comparison with the inner annular wall. This is likely to affect the strength of the recirculation zones, and the re-ignition of the flame along the outer shear layer. Furthermore, the thermal conductivity of the inner steel wall is approximately 10 times that of the outer quartz wall, potentially altering the recirculation zone temperature and re-ignition probability.

The synthetic experiment performed in Sect. 4 demonstrated that as the laser sheet and flame surface alignment becomes close to parallel, the accuracy of the method decreases. This can clearly be seen in the experimental results, when considering off-centre laser sheet locations, particularly those close to the flame edge. For example, in Fig. 9 the left and right hand side columns show slices through the flame close to the edge, and these show notably wider and more diffuse ${\overline{L}}$ distributions in comparison with the centre cross sections. The wider distributions are a result of the laser sheet and flame surface parallel alignment, which lowers the OH gradient magnitude, increasing the error associated with flame edge detection.

**Fig. 10:**
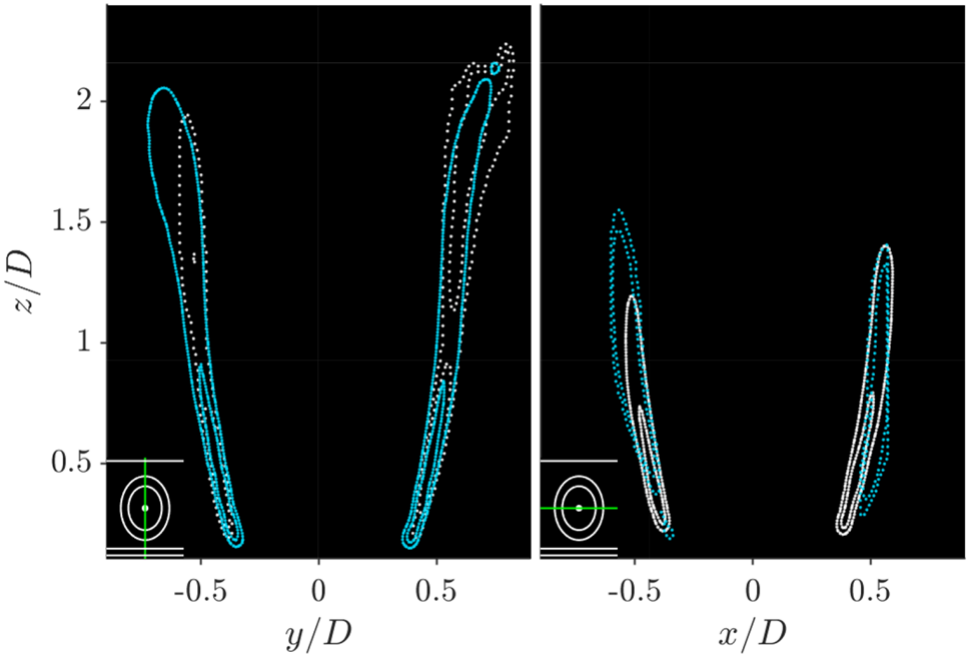
Comparison of centre line slices of both scan directions outlined as a contour plot (iso-value$=0.6\mathrm {FSD}_{max}$) capturing the flame shape of the represented slice, blue represents Scan YZ and white represents Scan XZ. The solid and dotted line represents the orthogonal and parallel slice (laser sheet to flame edge orientation) of each scan direction, respectively. Subplot represents position of the slices relative to the annular combustor geometry

However, despite these wider distributions, the flame shape is still captured relatively accurately. To demonstrate this the full three-dimensional FSD was evaluated, using the procedure outlined in Sect. 2. The central cross plane slice locations can then be compared by superimposing the two independent measurements from the different scan directions. Selecting the centre cross plane sample for this comparison means that from one scan direction the laser sheet is approximately orthogonal to the flame surface, while from the other direction it is approximately parallel, allowing the worst case alignment to be evaluated. The comparison is presented in Fig. 10, which shows superimposed contours corresponding to a single plane of the 3D reconstructed FSD distribution from the two scan directions. Given the variation in FSD intensity, the contour levels do not match each other. However, the similar contour shape distributions indicate that both scanning directions capture similar spatial distributions of FSD. The largest differences are observed in the $y-z$ plane, where the distribution close to the outer annular wall is notably wider when the reconstruction based on laser sheets aligned in the $x-z$ direction is considered. While not accounting entirely for this difference, the width of the distribution in the $x-z$ direction is affected by the size of the volume reconstructed, as data from laser sheet locations close to the flame edges had to be removed, due to the very low gradient values of the OH distribution, which prevented accurate flame edge detection. However, most of the differences are expected to arise from the differences in the OH gradients in the PLIF images from the two different scan directions, which the method has already been demonstrated to be sensitive towards. To further quantify this bias error, the entire FSD distribution in the measurement planes shown in Fig. 10 was integrated separately from the two separate scanning directions. This represents a worst case estimate of the bias error. It was found that the FSD in the $y-z$ and $x-z$ planes were under predicted by 68 and 51% by Scan XZ and Scan YZ, respectively. The larger FSD bias in the $y-z$ plane demonstrates that the underprediction is greater when the flame is close to the annular walls.

### Three-dimensional flame surface density

**Fig. 11:**
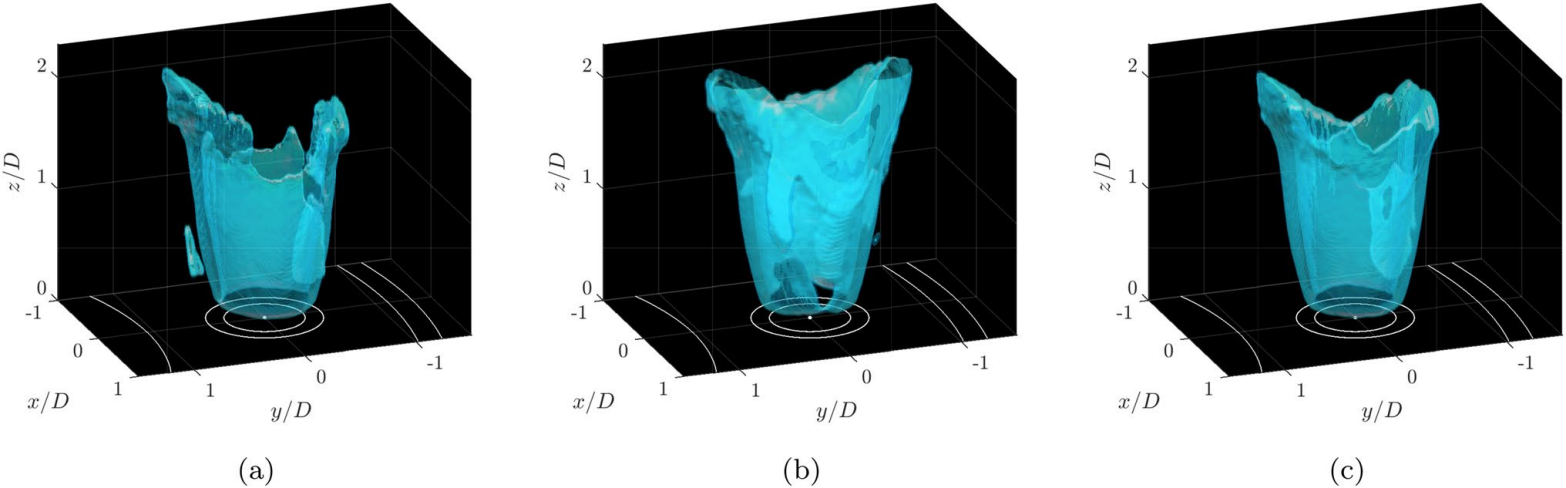
Isosurfaces of 3D FSD. (a) Scan XZ, (b) Scan YZ, (c) Combined Scan YZ and XZ. White lines show schematic representation of annular chamber and burner inlet. Isosurface level is the spatial average of the FSD

Isosurfaces of the 3D FSD are shown in Fig. 11, based on both the two independent scan directions, and the combination of these. The combined volume is reconstructed by averaging both volumes in each scan direction as described in Sect. 2 into a single discretised domain, therefore representing the linear addition of the two independent scans.

The flame shape is observed to vary in height with local azimuthal location, $\theta$, with the highest locations occurring close to the inner and outer annular walls. A similar flame shape can be observed in scans from both directions, albeit with portions of the flame surface poorly resolved in regions where the alignment of the laser sheet and flame surface were close to parallel. Therefore, a single scan is capable of broadly capturing the structure of the current flame, but care must be taken when interpreting the variation of FSD magnitude.

The independent measurements from the two scan directions raise the prospect of defining a correction factor to take account of the FSD magnitude change based on the laser sheet orientation. However, of the simple correction methods based on linear variation in the azimuthal polar or linear Cartesian directions, none were found that could adequately account for the observed intensity variations. However, by scanning from two separate directions, the weakness of the method to such bias error can be largely overcome, and the volumetric distribution in the combined reconstruction, shown on the right hand side of Fig. 11, produces a more uniform FSD distribution.

**Fig. 12:**
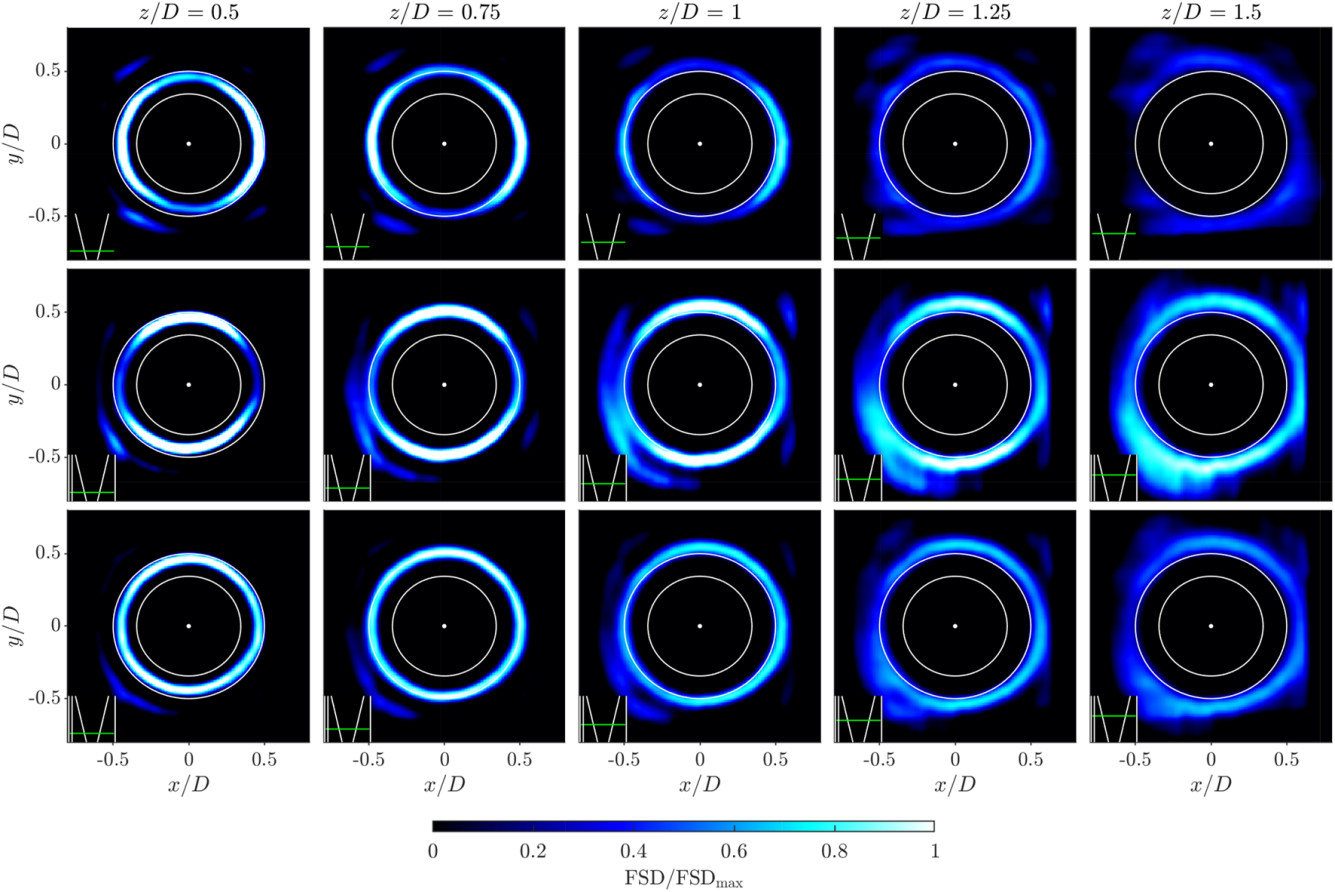
Slices of FSD in the $x-y$ plane for Scan XZ (top row), Scan YZ (middle row), and combined Scan XZ and YZ (bottom row). Slice location represented by green line in bottom left corner schematic

Flame asymmetry and orientation dependence of the reconstruction can be more accurately assessed by evaluating streamwise cross-sectional planes, as presented in Fig. 12. Three rows are presented, showing the two independent scan directions, and the combination of these. For each reconstructed volume $x-y$ plane slices are plotted for a range of downstream measurement locations, which is indicated in a small schematic in the lower left hand corner of each sub figure. Additionally, as a visual aid, the location of the circular bluff body and injection tube are show schematically, as solid white lines.

As described previously, the flame structure is described by all three rows. While the flame surface is poorly resolved in the top and middle row, when the laser sheets are almost parallel with the flame surface, the combination of these data sets reduces the underestimation of FSD significantly, and the flame structure is therefore best represented in the bottom row. Close to the dump plane, at $z/D=0.5$, the flame structure is almost axisymmetric, with a diameter of approximately *D*. At increasing distance above the dump plane the flame expands radially, and the flame brush thickens. The distribution of FSD also becomes more asymmetric with increasing height, with lower FSD intensity where the flame is closest to the neighbouring flames ($|x/D|>0.4$), as a result of the reduced flame length in these regions.

These cross plane slices clearly show the underestimation of the FSD due to the laser sheet alignment angle. In the upper row at $z/D=0.5$ the left and right hand sides of the FSD distribution are around twice the intensity of the top and bottom edges. Similarly, in the middle row at $z/D=0.5$ the top and bottom edges of the FSD distribution are much higher intensity than the left and right hand side.

It is also interesting to note that at the extremities of the flame ($|x/D|>0.5$, $|y/D|>0.5$) some flame elements are also captured in the outer shear layer. These flame elements appear only at the effective corners of the domain, and are likely a result of the complex recirculation zone pattern generated in the annular enclosure. This allows flame elements to be stabilised only at certain azimuthal locations, adding additional asymmetry to the flow. While asymmetry arising from the enclosure is clearly observed from the reconstructed FSD, asymmetry arising from other sources such as the grub screws, and flow conditioning from the plenum flow were not observed, meaning these are likely to play a more minor role in the symmetry of the mean flame structure.

### Integrated local heat release rate

**Fig. 13:**
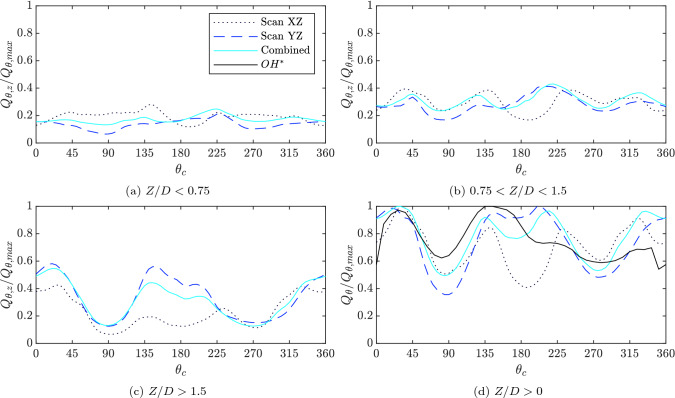
Heat release rate of azimuthal wedges over the range of upstream positions of the flame normalized by maximum flame heat release rate of the entire flame for Scan XZ - dotted line, Scan YZ - dashed line, and the combination of both - solid line

To provide a more quantitative assessment of the flame symmetry, the 3D heat release rate was integrated in wedge shaped regions (Eq. 2) over a range of downstream locations, and is presented in Fig. 13. The integrated regions were normalised by the maximum heat release rate of the wedges rotated around the entire flame, $Q_{\theta ,max}$, to assess the local heat release rate contribution to the total heat release rate.

The fluctuation of the heat release rate and the loss of symmetry increases with downstream distance. In Fig. 13a, the HRR is represented for the base of the flame ($Z/D < 0.75$). When considering the individual scan directions a decrease in the HRR which represents the loss of axisymmetry is observed at locations orthogonal to the scan direction, that is $\theta _c\approx 0^\circ$ and $\theta _c\approx ~90^\circ$ for Scan XZ and Scan YZ, respectively. However, the reconstruction of the flame based on both scan directions produces a fairly constant HRR, demonstrating the symmetry of the flame close to the exit of the injector. Similar symmetry features can be seen when integrating the flame over the middle region ($0.75< Z/D < 1.5$), as shown in Fig. 13b, and again the combined case eliminates some of the asymmetric variations in intensity. Small peaks are also present for all reconstructions that highlight locations corresponding to flame elements stabilised in the outer shear layer. These can seen at locations $\theta _c\approx ~45^\circ , 130^\circ , 220^\circ$ and $320^\circ$.

Larger departures from axisymmetry are visible in Fig. 13c, which shows the upper portion of the flame. The HRR decreases significantly at $\theta _c\approx ~90^\circ$ and $270^\circ$ corresponding to significant variations in flame length due to the presence of walls or neighbouring flames. The combined case is dominated by data from scan direction YZ since the near walls regions, which contribute most in this part of flame, were not well resolved in Scan XZ. The large asymmetry in this upper region contributes significantly to the total integrated distribution shown in Fig. 13d. Although regions with a greater amount of flame elements stabilised in the outer shear layer can still be seen, these features are less prominent in the overall integrated FSD. A comparison of integrated FSD and overhead OH$^*$ chemiluminescence (calculated from the overhead image shown in Fig. 7) is also presented in Fig. 13d. There is reasonable agreement between integrated OH$^*$ and FSD measurements in some regions, with the former picking up many of the peaks and dips associated with the azimuthal variation of HRR around the flame. However, the OH$^*$ prediction of HRR is lower close to the outer wall ($\theta _c\approx ~330^\circ$), which may be caused by the differing role of heat losses or flame quenching in the two methods.

## Conclusions

In this study a three-dimensional reconstruction of a non-swirling bluff body stabilised flame inside an annular combustor is performed using a novel variant of Scanning PLIF. Having access to the three-dimensional heat release rate distribution provides insight into the flame structure and asymmetry in response to asymmetric confinement.

A synthetic PLIF study was first conducted in order to optimise the experimental setup, and also to investigate the source of bias error in the reconstructions. The numerical study demonstrated the reliability of the edge detection, and the reconstruction procedure, as well as justifying the choice of experimental laser sheet thickness and sheet overlap parameters. A significant source of bias error was identified, both in simulations and in the later experiments, which occurs when the flame front and laser sheet approach parallel alignment. A similar source of error may be present to some degree in all PLIF experiments, requiring the careful interpretation of results.

To address this issue, experimental measurements were conducted from two orthogonal scanning directions, with the results analysed both separately and through a combined FSD calculation. Reconstructions from the different scanning directions were showed to resolve similar flame shapes, but tangentially aligned regions produced a bias error when determining the magnitude of FSD. However, combining data from two orthogonal scans, largely resolved this bias, returning largely axisymmetric distributions of the heat release rate.

Having access to the full three-dimensional heat release rate permitted a detailed assessment of spatial distribution of this. In this manner the asymmetry of a nominally axisymmetric flame subject to asymmetric confinement was been described, providing insights into the flame structure. The reconstruction demonstrated that the bluff-body flames stabilised in annular chambers are significant affected by the asymmetric confinement. The flame was longer when it was close to the annular walls in comparison to when it was close to a neighbouring flame, resulting in significant asymmetry.

This study outlined the importance of the laser sheet to flame edge relative orientation. Two laser sheet orientations were tested in this work, but in future work it be may be advantageous to consider other orientations, which minimise instants of parallel alignment; for example orienting the laser sheet parallel with the dump plane. This was not considered in this study due to the significant technical challenge it presents. The possibility of implementing a correction factor to overcome the FSD underestimation when using a single scanning direction should also be the focus of future developments. While multiple attempts at finding a suitable correction were attempted in the present work, a suitable correction method was not identified. A correction factor may have implications for other studies in which FSD is calculated on multiple planes of interest.

## Figures and Tables

**Fig. 14:**
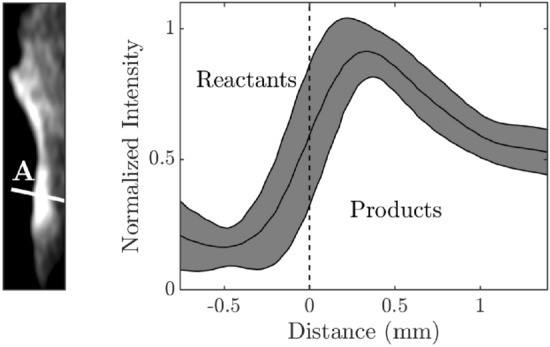
Experimental determination of a mean OH intensity profile in terms of normal distance from the flame front. Left: enlarged raw PLIF image with line, A, representing normal to flame front from which intensity distribution was determined. Right: Black line represents mean OH profile, shaded region represents standard deviation of the extracted edge profiles. Dashed line indicates identified flame front location

**Fig. 15:**
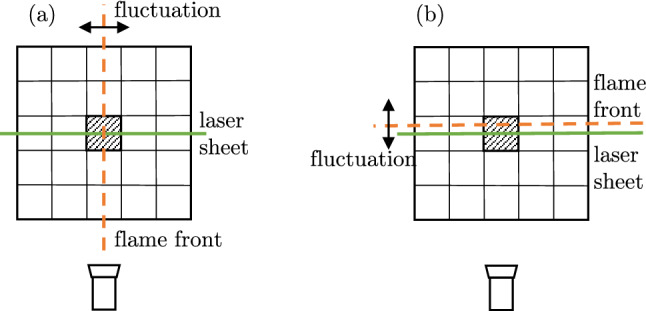
Schematic of laser sheet position and flame sheet **a** perpendicular **b** parallel in orientation viewed from above with grid lines representing the voxels of the domain

## Appendix 1: Experimental determination of OH distribution

The OH distribution captured through PLIF is a complex function of many parameters, such as fluorescence detector efficiency and gain, radical concentration, and excitation efficiency  (Kohse-Höinghaus 1994; Hassel and Linow 2000). While DNS has been used previously to simulate the OH distribution (Atkinson et al. 2014), in the present study a simplified approach is used. The volumetric OH distribution is determined by combining the ideal surface location for each snapshot with the experimentally measured OH distribution through a flame front. In addition to its simplicity, the advantage of this approach is that the important flame front intensity distribution in the synthetic images closely resembles that of the experiment.

The measured intensity distribution normal to the flame front at a single pixel location along the flame front was extracted close to the flame base, where a clear gradient distribution was present. Similar distributions were extracted from a set of 10000 images. A single laser sheet location was used for this purpose, which was aligned with the centre of the bluff body, thereby cutting through the centre of the flame. The intensity profiles were then averaged, as shown in Fig. 14.

## Appendix B Positional bias in determining flame locations

This paper introduced the biases produced when finding edges of the flame and its respective sensitivity to laser sheet thickness and image noise. However, another form of bias exists simply based on the laser sheet position and the flame front orientation. To better demonstrate this effect consider Fig. 15a, which represents an overhead view of an experimental setup where the laser sheet and flame front are perpendicular to each other. For simplicity assume that the flame front only fluctuates perpendicular to the laser sheet. If the actual occupancy rate in a single voxel of interest (represented as the shaded voxel) is $L_{act}$, then the measured occupancy rate, $L_{measured}$, would be equal to the actual since the fluctuations in flame location will always be captured on the camera as it intersects with the laser sheet perpendicularly. In contrast consider the orientation where the laser sheet and the flame front are parallel as represented in Fig. 15b. This arrangement would result in $L_{measured} = 0$ since the flame front either does not intersect with the laser sheet, or is parallel to it when intersecting, making the gradient between products and reactants indistinguishable. This simple demonstration suggests that there is no ideal laser sheet that can completely resolve the flame when the mean flame shape produces fluctuations that are parallel to the laser sheet. It is worth highlighting that such a bias will not just affect the present measurements, but given the highly turbulent nature of many flows of interest, such errors may be present but not reported in a wide range of studies.
